# Comment on a published new method in virucidal testing – T-25 method 

**DOI:** 10.3205/dgkh000638

**Published:** 2026-03-10

**Authors:** Ingeborg Schwebke, Johannes Blümel, Nils-Olaf Hübner, Janis A. Müller, Holger F. Rabenau, Eike Steinmann, Jochen Steinmann, Ingrid Rapp, Miranda Suchomel, Sven Reiche, Maren Eggers

**Affiliations:** 1 Expert Committee on Virus Disinfection of the German Association for the Control of Viral Diseases (DVV) e.V. and the Society for Virology (GfV) e.V., Aschaffenburg, Germany

## Comment

In October 2023, the T-25 method was published as a method for reducing cytotoxicity in virucidal testing of disinfectants in the journal *Heliyon: *v. Rheinbaben F, Köhnlein J, Schmidt N, Hildebrandt C, Werner S. To reduce cytotoxicity when testing the virucidal activity of chemical disinfectants and biocides: The “T-25 method” as an alternative to “large-volume-plating” Heliyon. 2023 Oct 9;9(10):e20728. DOI: 10.1016/j.heliyon.2023.e20728 [1].

As experts of the German DVV/GfV Virus Disinfection Commission, we are of the opinion that while this method is of interest, it requires further explanation and commentary. Unfortunately, the publication of a commentary in the journal *Heliyon* is not permissible. Therefore, the classification and evaluation of the contribution by Rheinbaben et al. is presented in the following form.

The German Association for the Control of Viral Diseases was established in the 1950s with the objective of combatting polio: In September 1953, Leading Medical Officers of the Federal German States and Free Cities met in Hamburg. The following three tasks were identified as priorities:

1. Researching the polio disease,
2. Finding effective and safe means of combating it, and
3. Helping those suffering from the consequences of the polio disease.

Consequently, the German Association for the Control of Viral Diseases (DVV) was founded on 31 August 1954 in Berlin. It was established as an interdisciplinary instrument for doctors and scientists in practice, clinics, research institutes, and health authorities. The initiative is endorsed by the Federal Ministry of Health, the health ministries of the federal states, and supported by scientific societies, and socially committed foundations and organizations. The DVV’s initial focus was on combating poliomyelitis in Germany. As the disease was brought closer to eradication and major advances in the fight against other viral diseases became apparent thanks to the development and consistent use of new vaccines, the association’s activities were expanded accordingly. Since then, the DVV is responsible for the provision of support to the public health service, the Federal Ministry of Health, the federal authorities such as the Robert Koch Institute as well as for the provision of support to foreign and international organizations in matters relating to infectious diseases, their prevention, detection, control, and diagnosis. The Commission for Virus Disinfection is a key component of the public health service, providing vital information to federal states. It is now a joint commission of the DVV and the German Society of Virology (GfV), so the commission’s main focus is disinfection against viral diseases. Thus, the Commission for Virus Disinfection follows new methods with great interest, recognizing their potential to standardize disinfectant efficacy testing. At the same time, in its role as an advisory body to the federal states, their healthcare systems, and federal authorities, the Commission must also assess how such methods should be classified and integrated into existing frameworks. This requires not only a careful consideration of the scientific and clinical benefits but also an awareness of possible limitations, risks, and regulatory implications. On the one hand, innovative methods may offer greater accuracy, efficiency, or accessibility. On the other hand, they may also involve uncertainties, technical challenges, or additional resource requirements. By weighing these advantages and disadvantages, the Commission ensures that recommendations are both forward-looking and aligned with the needs of healthcare systems and regulatory. Disinfectants contain active substances that can damage cells and thereby induce a cytopathic effect (CPE). This disinfectant-related CPE is often indistinguishable from the CPE caused by viral infection. However, in order to demonstrate the required reduction in viral infectivity by the disinfectant, it is essential to differentiate between cytotoxicity and true viral activity. This becomes particularly critical in experimental settings where high cytotoxicity coincides with low viral titers. In such cases, detoxification procedures are necessary to minimize cellular damage caused by the disinfectant, thereby enabling a reliable assessment of residual viral infectivity.

Rheinbaben et al. present a new method to reduce cytotoxicity when testing the virucidal activity of chemical disinfectants and biocides [1].

The products examined were based on chlorhexidine, octenidine or quaternary ammonium compounds and pose a major challenge for activity testing because of their cytotoxicity for the indicator cell cultures – even at low concentrations. Products containing these ingredients are often used for the antiseptic treatments of skin and mucous membranes. Such products can be medicinal products, medical devices or biocidal products, which have also been used against pandemic respiratory viruses such as SARS-CoV-2 or influenza to limit the spread of the viruses when necessary [2].

In the European standard EN 14476, designed for the testing of disinfectants and antiseptics, large-volume plating (LVP) is the recommended method for products with high cytotoxicity [3]. In the present study, the authors present the “T-25 method” using 25 cm^2^ culture flaks (T25) as an alternative to previously published methods such as the LVP method using multiwell plates [1], [4].

It is acknowledged that, for both the LVP and T-25 method, reducing cytotoxicity of test samples by suitable predilution of samples and investigation of large sample volumes may help to overcome the problems of cytotoxicity. Unfortunately, however, many aspects of the method presented by, Rheinbaben et al. remain unclear. 

In the following, we would like to discuss some of these aspects and data inconsistencies, which are important for drawing final conclusions and recommendations when applying this method.

In order to reliably compare the accuracy of LVP and T-25 methods, only those results can be thoroughly evaluated for which test data are available for both methods. Unfortunately, only 9 of the 22 tests, were performed with both the LVP and T-25 methods. In detail, these 9 tests reveals that residual virus was detected in only one test (Product #2). In this case, no differences were found between the outcome of both methods in which Product #2 showed no virucidal activity. In the remaining 8 comparable tests, no residual virus was detected by either method, so it is not possible to conclude at this time whether T-25 method or LVP is more accurate. Furthermore, there are no data available for the T-25 method regarding virus titers with or without product exposure. 

It is important to note that the T-25 method is currently used as a qualitative method for virus isolation in diagnostics. It requires a well-described mathematical basis when used as a quantitative method. In this publication, for the T-25 method, between one and three T25 cell culture flasks were used for each dilution. However, Tab. 1 does not indicate how many flasks were used to obtain the specific data. It appears, even if only one flask was used to determine the reduction, the Spearman/Kärber method [5], [6] was used for the calculation. 

Applying the Spearman/Kärber method [5], [6] under these circumstances is questionable. The result from only one flask would be very inaccurate. It is common to use a larger number of parallels (wells or flasks) – more than 6 – for such determination.

Although the work presented by Rheinbaben et al. contains interesting aspects, both the informative value and the validity of the work are limited due to the methodological weaknesses and ambiguities as well as lack of reporting. Important original data (e.g. titers, number of replicates, number of plates, etc.) are missing to objectively assess the significance of the T-25 method. It would be desirable for the authors of this letter and the scientific community to provide these data at a later stage, as otherwise the paper would be of limited use for reasons of practicability and reproducibility.

## Notes

### Authors’ ORCIDs

- Schwebke I: https://orcid.org/0009-0007-6708-5845
- Blümel J: https://orcid.org/0000-0001-7611-642X
- Hübner NO: https://orcid.org/0000-0002-6095-4936
- Müller JA: https://orcid.org/0000-0002-0347-416X
- Rabenau HF: https://orcid.org/0000-0001-9394-2176
- Steinmann E: https://orcid.org/0000-0002-3654-9965
- Steinmann J: https://orcid.org/0000-0002-0527-8840
- Rapp I: https://orcid.org/0009-0003-8408-1951
- Suchomel M: https://orcid.org/0000-0001-8758-9652
- Reiche S: https://orcid.org/0000-0001-7575-2483
- Eggers M: https://orcid.org/0000-0001-8485-9485

### Funding

Not applicable.

### Acknowledgments

Not applicable.

### Competing interests

The authors declare that they have neither financial nor non-financial competing interests.

The author Eggers M is an employee of the test laboratory Labor Prof. Dr. G. Enders MVZ GbR and declares that no conflicts of interest exist regarding the opinion presented here. 
